# The influence of induction speed on the frontal (processed) EEG

**DOI:** 10.1038/s41598-020-76323-8

**Published:** 2020-11-10

**Authors:** D. P. Obert, P. Sepúlveda, S. Kratzer, G. Schneider, M. Kreuzer

**Affiliations:** 1 Technical University of Munich, School of Medicine, Department of Anesthesiology and Intensive Care, Munich, Germany; 2 Department of Anesthesiology, Hospital Base San José, Osorno/Universidad Austral, Valdivia, Chile

**Keywords:** Neuroscience, Medical research

## Abstract

The intravenous injection of the anaesthetic propofol is clinical routine to induce loss of responsiveness (LOR). However, there are only a few studies investigating the influence of the injection rate on the frontal electroencephalogram (EEG) during LOR. Therefore, we focused on changes of the frontal EEG especially during this period. We included 18 patients which were randomly assigned to a slow or fast induction group and recorded the frontal EEG. Based on this data, we calculated the power spectral density, the band powers and band ratios. To analyse the behaviour of processed EEG parameters we calculated the beta ratio, the spectral entropy, and the spectral edge frequency. Due to the prolonged induction period in the slow injection group we were able to distinguish loss of responsiveness to verbal command (LOvR) from loss of responsiveness to painful stimulus (LOpR) whereas in the fast induction group we could not. At LOpR, we observed a higher relative alpha and beta power in the slow induction group while the relative power in the delta range was lower than in the fast induction group. When concentrating on the slow induction group the increase in relative alpha power pre-LOpR and even before LOvR indicated that frontal EEG patterns, which have been suggested as an indicator of unconsciousness, can develop before LOR. Further, LOvR was best reflected by an increase of the alpha to delta ratio, and LOpR was indicated by a decrease of the beta to alpha ratio. These findings highlight the different spectral properties of the EEG at various levels of responsiveness and underline the influence of the propofol injection rate on the frontal EEG during induction of general anesthesia.

## Introduction

Already in 1847, 1 year after the first successful demonstration of ether anaesthesia, John Snow described five stages of general anaesthesia—a state of amnesia, analgesia, unconsciousness, and immobility without harming the patient^1,2^. Almost 100 years ago, Arthur Guedel refined these observations and established the Guedel’s classification^3^ for ether anaesthesia. This classification consists of four stages: stage of analgesia, stage of excitement, surgical stage, and stage of respiratory paralysis. Guedel defined the second stage as follows: “*The loss of volition and consciousness inaugurates this stage which represents the period of excitation of most major cerebral and cerebellar centers as well as reflex centers, preceding their depression*.” The first systematic descriptions of thiopental induction showed some differences between intravenous and inhaled drugs^4^. Today general anaesthesia is mainly induced by bolus injection of an intravenous anaesthetic (mostly propofol). With this approach, the transition from wakefulness via the loss of consciousness (LOC) to anaesthetic levels adequate to facilitate mask ventilation or intubation and finally surgical intervention is a matter of seconds to minutes and the excitatory stage is either of short duration or cannot be observed^5,6^.

In the last decades there have been numerous studies and enormous efforts to correlate the LOC with changes in electroencephalographic (EEG) oscillations^7^. In daily clinical practice, anaesthesia induction typically leads to sudden changes in the EEG^8^. In case of slower induction the transformation in the EEG may be more transient^9^. In addition, the choice of propofol induction speed may also influence the underlying mechanisms that cause the LOC^10^.

Recent research showed that the way we lose conscious perception of the environment has cortical and subcortical origins and the state transition is reflected in the EEG. Cortical connectivity is crucial for the emergence of consciousness and its loss seems to present an important mechanism of anaesthetic induced LOC^11,12^. Still, monitors as used in daily practice focus on the frontal EEG, especially on its spectral composition. The most common anaesthetics cause a shift in the EEG towards slower oscillations with higher amplitudes, but there are differences among substances^13^. In general, the EEG under adequate general anaesthesia with propofol is slower with higher amplitudes and prominent alpha (8–12 Hz) and delta (0.5–4 Hz) oscillations^14,15^. Especially during slow anaesthesia induction with propofol, the EEG may traverse an episode with increased beta activity, termed ‘*paradoxical excitation*’ and subsequently delta and alpha oscillations evolve^16,17^.

Recent findings described patients who expressed these delta and alpha oscillations in the EEG suggested as signs of ‘*adequate anaesthesia*’, but were still responsive as assessed with the isolated forearm technique^18^. They were termed *black swans* with regard to Karl Popper^19^. Given that propofol is injected rather rapidly under circumstances of daily clinical practice the transition from awake to LOC is a highly dynamic process. Taking into account that even under these conditions we can observe responsive patients with a stereotypic LOC-related EEG pattern, this phenomenon may be more pronounced when propofol is injected slowly. As the period of loss of consciousness may also be prolonged during slow induction, there is a need for a clear definition of (un-)consciousness^20,21^. Unfortunately, (un-) consciousness can hardly be measured directly^22^. For this reason, (un-) responsiveness is often used as a proxy^20,23^. Since there are different levels of responsiveness and various scores to test for it, however, the applied stimulus should be taken into account when evaluating the loss of responsiveness^24–26^.

Here, we present findings from patients who received a very slow induction that lead to the suggestion that everyone can express spectral EEG features frontally, indicative of an unconscious patient. These features were observed at loss of responsiveness to verbal command (LOvR) and even more pronounced at the loss of responsiveness to painful stimulus (LOpR). We also compared the EEG of these patients to the EEG from patients with fast propofol induction, to highlight the EEG differences caused by induction velocity.

## Results

### Demographics

From our 9 patients with fast induction, we excluded three, because they developed EEG burst suppression in the induction phase. This burst suppression EEG has completely different EEG characteristics than non-burst suppression anaesthesia and would hence bias the results. The three excluded patients were 57, 57, and 61 years of age, i.e., the oldest ones in the fast induction group. The patients in the slow induction group were significantly older (p = 0.016; median: 48 years; min, max: 31, 62 years of age) than the remaining six patients of the fast induction group (27.5 (22, 44) years of age). There was no significant (p = 0.402) difference in the BMI between the patients with slow and fast induction 25.0 (21.6, 30.9) kg/m^2^ versus 23.9 (21.6, 27.2) kg/m^2^. The median duration until LOpR was 834 s (683 s, 1139 s) in the slow induction group which was significantly longer (p < 0.001) than in the rapid induction group (90 (72, 116) s). The median effect-site concentration for propofol at LOpR was 2.6 (2.1, 3.2) µg/ml for the patients with slow and 4.6 (4.4, 5.2) µg/ml (p = 0.01) for the patients with fast induction.

### Evaluation of the delay between LOvR and LOpR

We could not clearly separate the LOvR from the LOpR in the fast induction group. 7 out of 9 patients had the last response to verbal command within the same investigation interval as the last reaction to a painful stimulus. On the other hand, in the slow induction group we were able to discriminate between LOvR and LOpR since the median number of tests for responsiveness between LOvR and LOpR was 4 (0, 6), i.e., between 0 and 210 s.

### General spectral properties and differences in the beta ratio between patients with fast and slow induction

**Figure 1:**
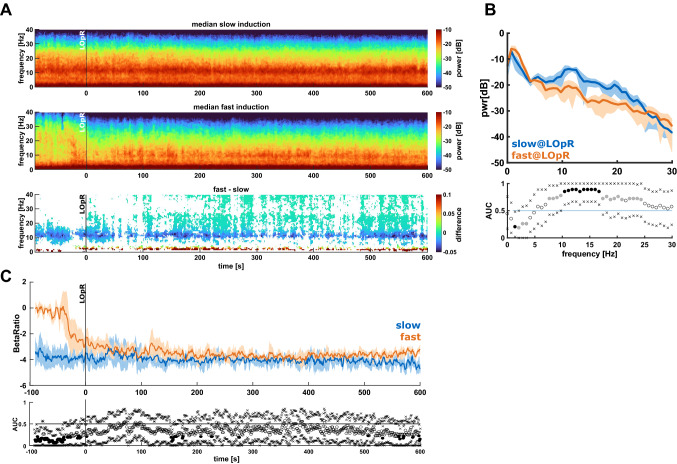
Spectral properties of patients with slow and fast propofol induction. Differences in the spectral EEG features between patients with fast and slow induction. (**A**) Density spectral array (DSA) of the normalized power spectral density (PSD) for patients with slow (top) and fast (centre) induction. The bottom plot presents the difference in power for each time and frequency between patients with fast and slow induction. Only pixels with a significant difference are displayed. For the patients with slow induction, strong alpha and delta activity was already present before loss of responsiveness to painful stimuli (LOpR), whereas in the patients with fast induction these patterns developed after LOpR. The difference plot revealed higher relative power in the delta range in patients with fast induction, whereas patients with slow induction had higher relative beta band starting around LOpR and higher relative alpha power in the entire observation period. (**B**) PSD plot showing the normalized spectral power for patients with slow (blue) and fast (orange) induction in combination with the area under the curve (AUC) and 95% confidence interval (CI) for each frequency for the 10 s after LOpR. Black dots in the AUC plot indicate significance and grey dots an AUC > 0.7. The “x” indicate the boundaries of the 95% CI. Patients with slow induction had less relative power in the delta range and higher relative power in the alpha and beta range. (**C**) The course of beta ratio in patients with fast induction showed a strong decrease before LOpR and it was significantly different from patients with slow induction before LOpR as indicated by the AUC and 95% CI presented in the lower graph.

In a first step we evaluated the differences in spectral EEG features between the patients with slow and fast induction. Figure 1A shows median density spectral array (DSA) derived from the normalized power spectral density (nPSD) for all patients of both induction groups as well as significant differences in the frequency ranges from 90 s before LOpR until 600 s after LOpR.

In the fast-slow difference plot (Fig. 1A 3rd row) only pixels are presented that returned a significant result (p < 0.05, Mann–Whitney U). The green and blue colours indicate significant higher relative power in the patients with slow induction and red colours indicate higher relative power in the patients with fast induction. The slowly induced patients had higher relative power in the alpha and beta range and lower relative power in the delta range. The onset of higher relative beta power was after LOpR, while we also observed the differences in relative alpha and delta power before LOpR. Figure 1B presenting the PSD at LOpR in the slow and fast induction group reflects the lower delta power, but higher alpha and beta power in the patients with slow induction. This highlights a difference in EEG characteristics at LOpR, dependent on induction speed.

We calculated the beta ratio as a proxy for the bispectral index to evaluate possible differences in the parameter between patients with slow and fast induction. Figure 1C presents the trend data for the beta ratio. Here as well different dynamics towards the LOpR can be observed. While the patients with fast induction followed the expected course of a decreasing beta ratio^27^, the patients with slow induction did not as in this group we observed a low beta ratio already before LOpR. This finding is statistically confirmed by a significant higher beta ratio in the fast induction group until LOpR (and some episodes with lower beta ratio in the patients with slow induction after LOpR).

### Analysis of the slow induction at loss of responsiveness to verbal or painful stimulation

#### General spectral EEG properties

**Figure 2:**
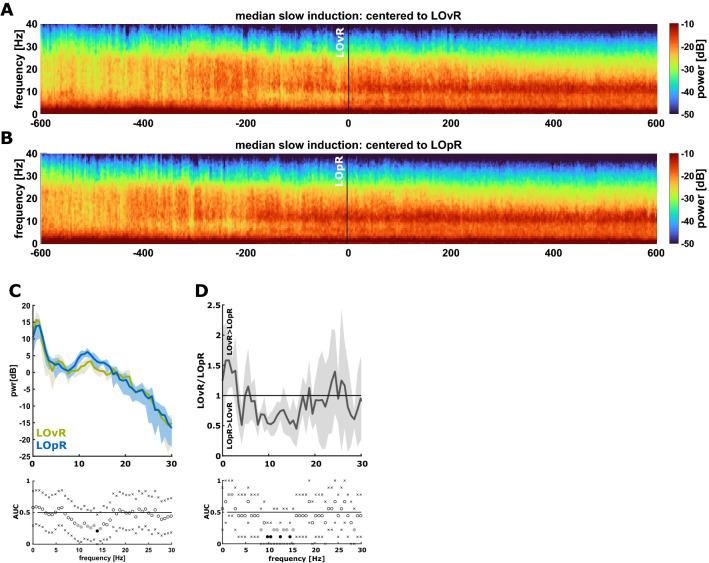
DSA and PSD of patients with slow induction at loss of responsiveness to verbal command vs. painful stimulus. Differences in the spectral EEG features in patients with slow induction between the loss of responsiveness to verbal (LOvR) or painful (LOpR) stimulation. (**A**) Density spectral array (DSA) of the normalized power spectral density (nPSD) for patients undergoing slow induction centred to the LOvR. The development of the characteristic alpha and delta dominant power indicative of an anaesthesia EEG pattern develops prior to LOvR. (**B**) DSA of the nPSD for patients undergoing slow induction centred to the LOpR. When centred to LOpR the increase in EEG alpha-band power around 10 Hz is more pronounced as if centred to LOvR as in (**A**). (**C**) PSD plot showing the normalized spectral power for patients at LOvR (green) and LOpR (blue) in combination with the area under the curve and 95% confidence interval (CI) for each frequency for the 10 s after LOR. Black dots indicate significance and grey dots an AUC > 0.7. At LOpR patients had higher relative power in the 10–15 Hz range. The “x” indicate the boundaries of the 95% CI. (**D**) Ratio of the spectral power between LOvR and LOpR. The plot highlights that the power in the 10–15 Hz frequency band is significantly higher at LOpR indicated by the black and gray dots in the AUC plot.

Because of the slow induction we were able to separately evaluate the EEG dynamics for the LOvR and LOpR. Figure 2 presents the median DSA plots for all patients from the slow induction group, either centred around LOvR (Fig. 2A) or LOpR (Fig. 2B). In both cases, we observed strong delta activity throughout the observation period and a visual identifiable onset of alpha oscillatory activity before LOvR and LOpR. The comparison of the nPSD at LOvR versus LOpR revealed a significantly higher relative power in the 10–15 Hz range at LOpR (Fig. 2C). The within patient change of EEG power from LOvR to LOpR confirmed these results. The EEG significantly increased in the EEG alpha-band range towards LOpR as it decreased in a clinically relevant fashion (AUC > 0.7) in the low (delta) frequencies (Fig. 2D).

#### Band power properties

**Figure 3:**
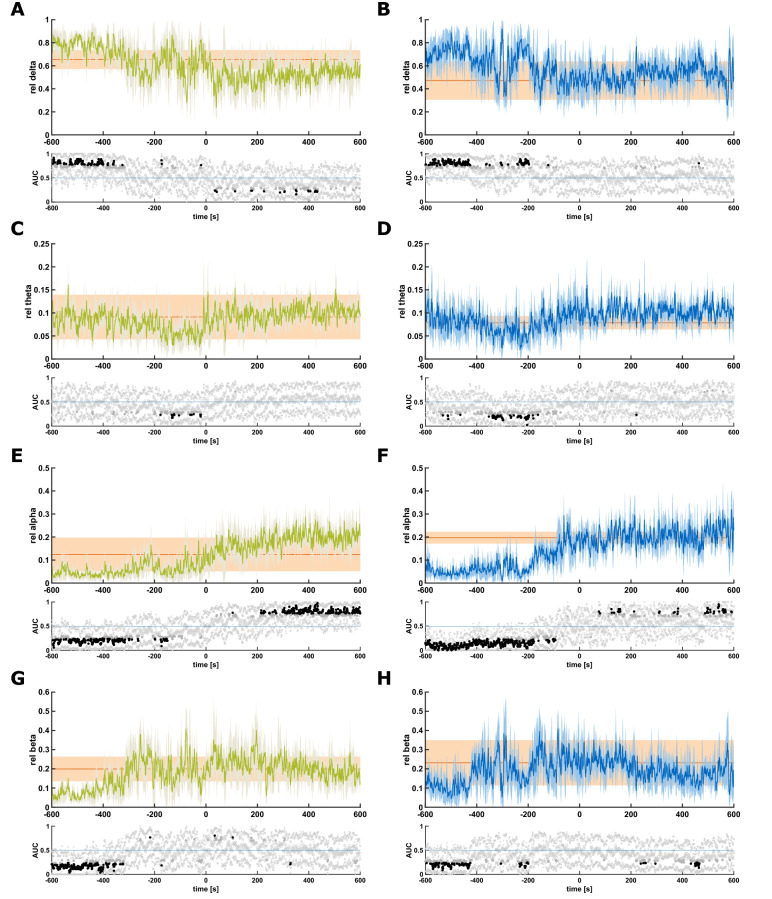
Relative band powers of patients with slow induction at loss of responsiveness to verbal command and painful stimulus. Course of the relative band powers for the 10 min before loss of responsiveness to verbal command (LOvR) in green and loss of responsiveness to painful stimulus (LOpR) in blue. The orange shading indicates the 95% confidence interval of the particular frequency band considering the first 10 s after LOvR or LOpR. The lower plots display the area under the curve. Black dots indicate significance. The “x” indicate the boundaries of the 95% CI. (**A**) The relative delta band power was significantly higher until around 300 s to 400 s before LOvR. The relative delta power decreased after LOvR. (**B**) The relative delta band power was significantly higher until around 200 s before LOpR, but did not decrease further after LOpR. (**C**) The relative theta band power was significantly lower for a short period before LOvR. (**D**) The relative theta band power was significantly lower for a short period before LOpR. (**E**) The relative alpha band power was significantly lower until around 3 min before LOvR and significantly higher after around 3 to 4 min after LOvR. (**F**) The relative alpha band power was significantly lower until around 2–3 min before LOpR and continued to increase after LOpR. (**G**) The relative beta power was significantly lower till around 300 s before LOvR. For the remainder of the observation period, it was not significantly different compared to the LOvR. (**H**) The relative beta power was significantly lower till around 200 s before LOpR and again starting after around 250 s 450 s LOpR.

In the next step we analysed the relative EEG band-power in the various frequency bands around the LOvR and LOpR (Fig. 3). The figure also contains the statistical evaluation of the band power at the different time points compared to the EEG derived from 10 s before to 10 s after LOvR and LOpR, respectively. In the patients with slow induction, the relative delta power was significantly higher around 360 s before LOvR and 200 s before LOpR. In the course until 10 min after LOvR and LoPR the relative delta power did not change significantly (Fig. 3A,B). We found the least changes in the theta-band. Only for the LOpR we found a significantly lower relative theta power from around 400 s to 200 s before LOpR (Fig. 3C,D). The relative alpha power was significantly lower until around three minutes before LOvR and two minutes before LOpR. In both cases, the relative alpha power continued to increase throughout the observation period. Especially for the LOvR we found a significantly higher relative alpha power around 200 s after LOvR. This increase was not as pronounced for LOpR (Fig. 3E,F). The relative beta power was significantly lower until around 300 s before LOvR and around 200 s before LOpR (Fig. 3G,H). For the remaining observation period there were no significant changes. This may be indicative of the paradoxical excitation period during the transition phase into unconsciousness that lasts for several minutes when anaesthesia is slowly induced.

#### Processed EEG properties

**Figure 4:**
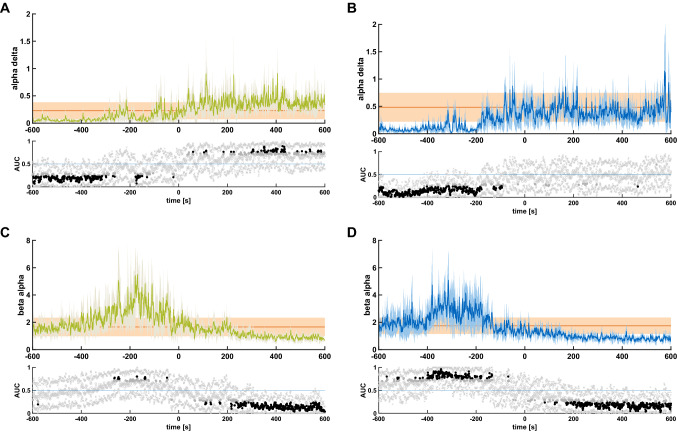
Band power ratios of patients with slow induction at loss of responsiveness to verbal command and painful stimulus. Course of the alpha delta and beta alpha ratio for the 10 min before loss of responsiveness to verbal command (LOvR) in green and loss of responsiveness to painful stimulus (LOpR) in blue. The orange shading indicates the 95% confidence interval of the particular band power ratio considering the first 10 s after LOvR or LOpR. The lower plots display the area under the curve. Black dots indicate significance. The “x” indicate the boundaries of the 95% CI. (**A**) The alpha to delta ratio was significantly lower till 3 min before LOvR and significantly higher around 5 min after LOvR. (**B**) The alpha to delta ratio was significantly lower till 2–3 min before LOpR, but did not increase significantly afterwards. (**C**) The beta to alpha ratio was significantly higher 250–150 s before LOvR and significantly lower around 3 min after LOvR. (**D**) The beta to alpha ratio was significantly higher until around 150 s before LOpR and significantly lower around 3 min after LOvR.

The calculation of the alpha to delta ratio showed a significant lower ratio till until around 3 min before the LOvR and a significant higher ratio starting around one minute after LOvR (Fig. 4A). On the other hand, for LOpR, it was significantly lower till around 90 s before LOpR but did not increase significantly afterwards (Fig. 4B). The beta to alpha ratio was significantly higher until ~ 150 s before LOpR and decreased significantly around 100 s after LOpR. The results for LOvR were similar but the pre-LOvR increase was not as pronounced as for LOpR (Fig. 4C,D). Figure S1 contains the trends for the different processed EEG parameters.

#### Visualization of the trajectories using the PCA algorithm

**Figure 5:**
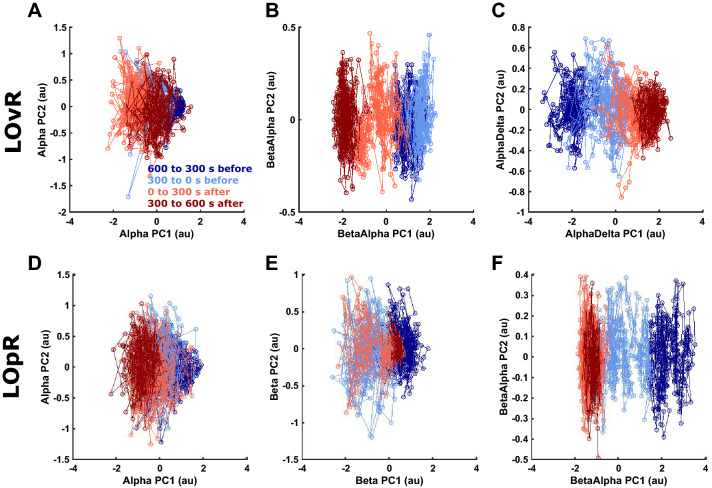
Visualization of the trajectories of patients with slow induction at loss of responsiveness to verbal command and painful stimulus using the PCA algorithm. Trajectories of different EEG band powers and band power ratios in the principle component (PCA) space constructed by the first two PCAs during loss of responsiveness to verbal command (LOvR) and loss of responsiveness to painful stimulus (LOpR). The blue to red colours indicate the time period relative to LOvR/LOpR starting with dark blue for the 10 min to 5 min episode before LOvR/LOpR until the 5 min to 10 min episode in dark red. (**A**) LOvR alpha: The colours change in a directional way, but with substantial overlap. (**B**) LOvR beta to alpha ratio: The colours change in a directional way with some overlap in the blue (pre-LOvR) colors. (**C**) LOvR alpha to delta ratio: The colours change in a directional way. (**D**) LOpR alpha: The colours change in a directional way, but with substantial overlap, especially in the red colours (post LOpR). (**E**) LOpR beta: The colours change in a directional way, but with substantial overlap, of the light blue and light red colour indicating the transient increase of beta power around LOpR. (**F**) LOpR beta to alpha ratio: The colours change in a directional way with almost completely overlapping red colours.

The visualization of the trajectories using the principle component analysis (PCA) algorithm in Fig. 5 and Supplemental Fig. 2 of the different relative band powers and ratios in the PCA space highlights the performance of the different parameters to separate the different episodes. The better the colours are separated in these plots, the better the transition can be tracked. We found the relative alpha power, the alpha to delta ratio, and the beta to alpha ratio to track the LOvR the best (Fig. 5A–C). For LOpR the relative alpha, relative beta, and beta alpha ratio showed the best separation (Fig. 5D–F). We present the PCAs for the other parameters as supplemental Figure S2.

#### Performance of various (processed) EEG parameters

**Figure 6:**
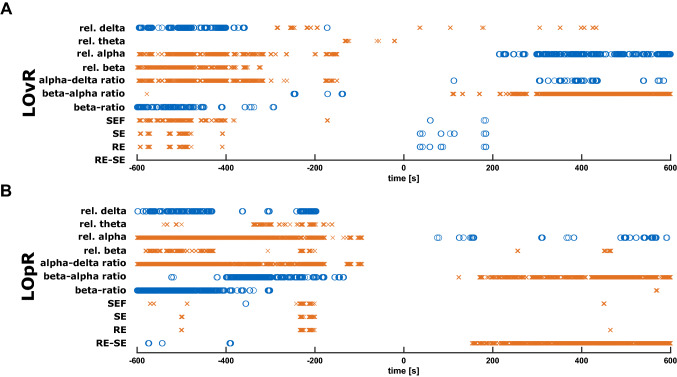
Performance of different EEG parameters to detect loss of responsiveness to verbal command and painful stimulus. This plot displays the performance of various (processed) EEG parameters to detect loss of responsiveness to verbal command (LOvR) and painful stimulus (LOpR). A change of colours around the LOvR/LOpR indicates a significant in-/decrease of the parameter when compared to the parameter at LOvR/LOpR. (**A**) LOvR: The relative alpha power and the alpha to delta ratio show a steady change throughout the transition. The beta to alpha ratio also shows the change in parameter, but less pronounced. (**B**) LOpR: The relative alpha power and the beta to alpha ratio show a steady change throughout the transition.

We calculated various (processed) EEG parameters and evaluated their performance to detect LOvR (Fig. 6A) and LOpR (Fig. 6B). A change of colours from pre-LOvR (pre-LOpR) to post-LOvR (post-LOpR) resembles a significant in-/decrease of the parameter. Orange indicates a significant lower value and blue a significant higher value when compared to the averaged value from − 10  to 10 s around LOvR/LOpR. Confirming the results of the PCA, relative alpha power and alpha to delta ratio seemed to be the most accurate parameters to detect LOvR. The best performance in tracking LOpR showed relative alpha power and the beta to alpha ratio. However, none of the evaluated parameters was able to detect LOR precisely as significant changes only occurred in a time period of 90 s around LOR.

## Discussion

With our analyses we are able to describe differences in the EEG dynamics during state transitions into unconsciousness that were triggered by either a fast or a slow anaesthesia induction. We used the FOUR score to track the transition into unconsciousness. This allowed for a comparison of the EEG characteristics around the LOpR between the slow and fast induction group. Because of the rapid dynamics during fast induction we could not clearly separate LOvR and LOpR. But we were able to perform separate analyses for the slow induction group allowing the distinction of these two anaesthetic stages as LOvR rather reflects (deep) sedation and LOpR indicates general anaesthesia^28^.

### Basic differences in the EEG between fast and slow anaesthesia induction

Our results show that the patients with slow induction had visually different transition DSAs with significant differences in the delta and alpha band range before the LOpR and additional significant differences in the beta frequencies after the LOpR. The use of the beta ratio as a proxy for intraoperative EEG monitoring suggests a significant difference in the trend of the “depth of anaesthesia” indices during anaesthesia induction that is dependent on induction speed. These observed differences may be due to a difference in the underlying mechanisms during fast or slow anaesthesia induction^10^. Sepúlveda et al. suggested a “top down” phenomenon with preserved brain stem functions at LOpR during slow induction^10^. This is in line with previous publications describing a primary impairment of the cortical functions at low propofol concentrations^29^. Another indicator of the distinct differences between a slow and a fast anaesthesia induction presents the occurrence of EEG burst suppression only in the fast induction group. The observation that three patients in the fast induction group had EEG burst suppression may suggest a general “deeper” level of anaesthesia in the patients with fast induction caused by an overshoot of the anaesthetic. These three patients were the oldest participants in the group underlining the higher risk of older patients for burst suppression^30^. Another indicator for such an overshoot is the generation of slow delta waves and the absence of alpha oscillatory activity in the EEG. Alpha seems to give way to delta oscillations at these “deeper” stages and a collapse of alpha activity may even be a predictor for nearing the stage of EEG burst suppression^31^. This transient overshoot is also reflected with very low depth of anaesthesia indices for a short period of time during anesthesia induction^32,33^. We refrained from further investigating the differences in the EEG between fast and slow induction, because we would have to delve deeper into the pharmacokinetics and pharmacodynamics during the transition and at least for a fast induction procedure these cannot be modelled properly^34,35^.

Hence, we focused on the EEG dynamics during the slow anaesthesia induction. The first finding to mention is that we could not significantly distinguish the EEG features observed at the LOvR or LOpR from the EEG features minutes before these events. For instance, we found an increase in alpha oscillatory activity already several minutes prior to LOvR or LOpR.

### Clinical relevance of alpha oscillatory activity during general anaesthesia

Strong oscillatory power in the alpha band of the frontal EEG may indicate a level of *adequate anaesthesia*^36–38^. Our observation of increased alpha band power prior to LOvR and LOpR indicates that these patterns can also develop in a responsive patient if the state transition is slow enough. Former research described patients that showed these EEG patterns of adequate anaesthesia, but were still responsive. The authors termed them “black swans”^18^. With our data we can show that the observation of this phenomenon depends on induction speed and that everyone may become a *black swan* in case of slow induction. A possible explanation may be found in the proposed mechanisms of intraoperative alpha activity. These anaesthetic-induced alpha oscillations seem to develop through anaesthetic action on the activity in the cortico-thalamo-cortical loop^39,40^ and reflect an impairment in information processing between these areas caused by synchronized volleys of neural bursts^41^. On the molecular level anaesthesia-related alpha oscillations develop as a result of a hyperpolarization of thalamocortical relay cells^42^. In the case of propofol, the hyperpolarization is induced by enhanced GABAergic inhibition of the thalamus itself (via enhanced GABAergic input from the reticular thalamic nucleus) and/or reduced excitatory input from corticothalamic projections^43^. At least in rats it has been shown that the effect of propofol on neuronal acitivity patterns is more pronounced in the thalamus than in the cortex^44^. This finding points towards a higher sensitivity of the thalamus to propofol compared to the cortex. Hence under the condition when the effect site concentration of propofol rises slowly the thalamus might be hyperpolarized and generate alpha oscillations whereas within the cortex the concentration is not high enough to induce LOvR or LOpR. When comparing the spectral EEG properties at LOvR and LOpR, we found higher alpha band power at LOpR. This finding confirms previous observations from Supp et al., who described an inverse correlation of alpha power and level of consciousness and is in line with the proposed increase in alpha power in the frontal cortex during general anaesthesia^14,45,46^. Modelling work suggests a slowing of cortical activity, which causes the oscillations in thalamocortical loops in the alpha band to synchronize^47^.

We did not observe the assumed direct correlation of LOvR onset and a diffuse increase in the power in the (low) beta and (high) alpha range^47^ or the delta-alpha ratio that seems to signal unconsciousness^46^. While all these patterns develop throughout anaesthesia induction, the slow induction paradigm may help to further understand the temporal dynamics in more detail. Our results point towards propofol induced effects on these oscillations that develop up to minutes before LOvR and LOpR. Again, these results are in line with the described cases of patients who expressed a peak in the PSD in the alpha range during anaesthesia induction with propofol thought to be indicative of unresponsiveness. Nevertheless, these patients showed a volitional response^18^ in contrast to our verbal command/painful stimulus following the FOUR score protocol. Another interesting observation was the increasing beta oscillatory activity during slow induction.

### Increased EEG beta power during slow anaesthesia induction

When inducing a patient with propofol, the EEG changes to faster oscillatory rhythm which causes a transient increase in the beta band power^17,48^. This episode is called *paradoxical excitation*^16^ or *beta-buzz*^49^. Modelling work on the mechanisms of the paradoxical excitation describe that this phenomenon may be caused by interneuron antisynchrony^50^ and/or by propofol causing a delay in transmission between cortical and thalamic structures^49^. In humans, findings also show that the effect on brain metabolism is similar in the thalamus and the cortex^51^. We also want to state that these proposed changes are different from events that cause increased frontal beta during wakefulness, for instance during the task of action stopping^52^. In our data, the beta power did not significantly decrease after LOvR and LOpR for some minutes. Hence, the paradoxical excitation phase may be prolonged in case of slow induction. Because the change in the EEG during this period resembles an ‘awakening’ the cognitive state during this stage should be evaluated in more detail in the future. For instance, regarding the question if the patients are really unconscious or transverse through a state of ‘disconnected consciousness’.

We also calculated EEG-band ratios and used the concept of PCA to evaluate what parameter may best track the LOvR and LOpR during slow induction.

### Tracking the slow transition

Based on the results from the trends of the relative EEG band power and the ratios of various band powers we evaluated their capability of tracking the transition into unresponsiveness. We would like to note that we did not focus on the correct detection of the time point of the LOvR and LOpR event, but on the course of the trend data, i.e., if it steadily increased or decreased during the transition. For LOvR, we found the relative alpha power and the alpha to delta ratio to show a steady increasing course. For LOpR it was also the relative alpha power and the beta to alpha ratio. The beta to alpha ratio had the least overlap as visualized in the PCA plots. All other parameters including the processed EEG parameters did not steadily increase nor decrease during the slow induction with the LOvR and LOpR events. The beta to alpha ratio seems to present a good way of tracking the LOpR, while the alpha to delta ratio seems good for the LOvR during slow induction, although with a lag as well. The high beta to alpha ratio before LOpR seems to be driven by the already increased beta power with alpha being lower than after LOpR. With ongoing time, the beta power stays about the same, but as the alpha power slowly increases this causes the beta to alpha ratio to decrease. Especially after the LOpR, the beta to alpha ratio presents the only tested parameter that remained significantly lower to LOpR in a stable fashion. For the LOvR, the alpha to delta ratio seems to work best, because it combines the quite steady decrease in relative delta and increase of relative alpha band power during the LOvR transition.

Our results add to the discussion regarding the role of the frontal cortex (alone) as anatomical area for the loss of responsiveness being separated into LOvR and LOpR in our analyses. While electrodes placed on a patient’s forehead are easy and quickly to apply for perioperative monitoring, findings from previous studies suggested e.g. the inclusion of parietal electrode positions^53^ as well to monitor the loss of information transfer between frontal and parietal cortices^11,12^. In non-anaesthesia studies using imaging approaches, a “*posterior hot zone*” was identified that seems to correlate with the contents of consciousness^54^. Hence, despite its practicability for clinical monitoring the forehead may not present the best electrode location to track (anaesthetic-induced) changes in consciousness. The performance of our parameters that are also used in clinical patient monitoring confirm this suggestion. As especially in the elderly—a patient group with higher risk for intraoperative awareness^55,56^—propofol should be administered slowly to avoid hypotension and cardiac depression, the inability of these parameters to track the slowly induced loss of responsiveness is worrisome^57,58^. Therefore, our results highlight the need of an optimization of (processed) EEG parameters to accurately detect loss of responsiveness during slow induction of general anaesthesia.

### Limitations

We could only analyse frontal EEG recordings. In order to evaluate spatiotemporal EEG dynamics during slow anaesthesia induction, a multi-channel EEG montage is necessary. Hence, the question of where does the EEG reflect the LOpR best has to be answered in future studies. Our EEG recordings were limited to frequencies up to 44 Hz. Since some of the processed EEG parameters we used include frequencies up to 47 Hz in the monitors, our results may not completely mimic the reaction of a monitoring device during slow induction. Our patient collective was rather young, so we cannot describe EEG changes during slow induction in the (very) old. From a clinical point of view, the fast induction (2 mg/kg body weight in 40 s) might have been on the very fast side and may not fully represent clinical routine. However, the standard regimen for induction also varies between hospitals and anaesthesiologists as some titrate the dosage manually and others use TCI mode. As this was a retrospective analysis we did not have any chance to influence the design but for future studies one might should adjust the TCI settings.

## Conclusion

With our results we could show that the EEG dynamics are different between patients that receive a fast or slow anaesthesia induction with propofol. When anaesthesia is slowly induced, frontal EEG patterns thought to correlate with an adequate level of anaesthesia evolve up to minutes before the patients lost responsiveness to verbal or painful stimulation. The beta activation known as paradoxical excitation persisted until minutes after the loss of responsiveness. The parameters best reflecting the loss of responsiveness during slow induction seem to be the delta to alpha ratio for LOvR and the beta to alpha ratio for LOpR. Our results also indicate that an optimization of processed EEG monitoring is necessary to reliably track the loss of responsiveness during slow induction.

## Methods

### Study approval and exclusion criteria

We based our analyses on EEG data from a blinded randomized single centre clinical trial that was approved by the Ethics Committee of the Clínica Alemana—Universidad del Desarrollo. The study was conducted in accordance with the Declaration of Helsinki. All patients signed informed consent prior to participate in the study (Clinical Trials register NCT03140982). Primary results were published earlier by Sepúlveda et al.^10^.

We included 18 adult patients from 18 to 65 years who were scheduled for elective non-neurologic, non-cardiac surgery. These patients had an ASA I-II status, had not received premedication, and presented with no history of neurological disease as well as with a normal clinical neurologic examination result. We excluded patients with a history of substance or alcohol abuse, documented adverse reactions to propofol and neurotropic drugs within 48 h prior to study procedures. All eligible subjects we randomly assigned in a 1:1 ratio to either a fast induction or slow induction group.

All analyses are based on 9 patients with slow induction and 6 patients with fast induction. Three patients with rapid induction were excluded from our analyses due to EEG burst suppression.

### Anaesthesia protocol

The subjects assigned to the fast induction group received propofol with target-controlled infusion (TCI) based on the Marsh model (ke0 1.21 min^−1^) at a calculated effect site target of 5.4 µg/ml (EC95 for propofol-induced LOC)^59^. The patients assigned to the slow induction group received propofol at a rate of 10 mg/kg/h until LOpR. Thereafter, the mode of propofol administration was the changed to TCI, using the effect-site concentration at loss of responsiveness to painful stimulus value as a target. In both groups, the infusion was maintained for 10 min without surgical stimulus. As TCI pump the Primea Orchestra by Fresenius-Kabi (Germany) was used. Data from effect-site concentration at LOpR were obtained from those calculated by the TCI pump.

### Clinical assessment of the loss of response to verbal and painful stimulation

LOR was evaluated by a neurologist before the start of the propofol infusion and every 30 s thereafter using the Full Outline of UnResponsiveness (FOUR) Score^26,60^. We determined LOvR as last point in time with a reaction of the eyelids to loud voice and we defined LOpR as was the absolute absence of ocular opening and the absence of responses to standardised verbal, tactile and painful stimuli (trapezius muscle pressure). To determine the latency between LOvR and LOpR we counted the number of tests at which the patient did not response to loud verbal commands but to painful stimuli.

### EEG pre-processing and analysis

We used EEG data from each patient from four frontal channels recorded with electrodes placed on the forehead during the whole surgical procedure using a SEDLine monitor (Masimo Corporation, Irvine, CA). Therefore, we used the custom, single-use SEDLine EEG strip that consists of six gel electrodes. The detailed sensor layout can be found in the SEDLine’s “Operator’s Manual”^61^. The electrode strips were placed as recommended. During recording the impedance levels were kept within the acceptable range as specified by the manufacturer. The recordings were conducted with two different sampling rates, i.e., 89 Hz or 178 Hz because the sample rate is dependent on the feed speed of the SEDLine’s EEG display^62^. To adjust for the differences in sample rate, we low-pass filtered all EEG recordings to the 44 Hz range and down-sampled the 178 Hz data to 89 Hz.

We visually inspected the EEG of each patient for the occurrence of EEG burst suppression (BSUPP), a pattern that indicates extensively deep levels of anaesthesia with EEG characteristics not comparable to the slow wave anaesthesia state^16,63^. We defined two observation periods. For the evaluation of differences in the EEG between patients with fast and slow induction, we focused on the period from 90 s before LOpR until 600 s after LOpR. For the investigation of the EEG recorded from the patients with slow induction only we extended the period to 600 s before until 600 s after LOpR.

In order to evaluate the EEG dynamics throughout the observation period, we calculated the power spectral density (PSD) using the *pwelch* function with a frequency resolution of 0.7 Hz for 5 s EEG segments with a 4 s overlap. We also calculated the nPSD, i.e., the PSD divided by its total power in the 0.7 to 30 Hz range. We had to choose this approach because the SEDLine EEG recording showed differences in amplitude scaling^62^. We display the averaged courses of the nPSD over time as density spectral arrays (DSA). Based on the spectral power information provided by the PSD and DSA we calculated a set of parameters. We calculated the relative delta (0.7–4.2 Hz), relative theta (4.2–7.7 Hz), relative alpha (7.7–12.5 Hz), and relative beta band (12.5–25.1 Hz) power as well as the alpha to delta and beta to alpha ratio. In order to reflect the possible index behaviour of processed EEG devices we calculated the beta-ratio, a sub-parameter of the bispectral index^27^, the spectral entropy (SpEnt) as used in the Entropy Module as well as the spectral edge frequency 95 (SEF95) also presenting a sub-parameter used in these devices. The beta ratio is defined as beta ratio = log((P30-47 Hz)/(P11-20 Hz)) and it is the parameter most strongly contributing to the bispectral index during light sedation^27^. The SpEnt is the Shannon entropy of the power spectrum within a defined frequency range, e.g., 0.8–32 Hz for the state entropy and 0.8–47 Hz for the response entropy^64^. We calculated the beta ratio and the spectral entropy as a proxy for the response entropy for a frequency range up to 44 Hz only because of our low sample rate. The SEF95 is a parameter displayed by the SEDLine monitor (Masimo Corporation, Irvine, CA, USA). The SEF95 is the frequency below which 95% of the EEG power is located^65^. We used the 0.7 to 32 Hz range for SEF95 calculation. For all analyses we used MATLAB 2017b (The Mathworks, Natick, MA). In order to visualize the EEG band power behaviour, we plotted the trajectories of the induction phase in the principle component (PCA) space constructed by the first two PCAs. For PCA calculation we used the MATLAB *pca* function. To add the trajectory to the plot we used the MATLAB *rainbow* function.

### Statistical analysis

For the evaluation of differences in the DSA, we used the Mann–Whitney U test. We did not correct for multiple comparisons and only discuss ‘clusters’ of significant results in the differences in spectral power over time (Fig. 1A 3rd row)^66^. The defined cluster size was 3 s × 2.1 Hz, i.e., 3 × 3 pixel. For the comparison between EEG parameters derived from the patients with fast or slow induction we calculated *area under the receiver operating curve* (AUC) with 10 k-fold bootstrapped confidence intervals for each 5 s section. We calculated the AUC with the MES toolbox^67^. In order to avoid detection of false positives we only considered time spans with at least three AUC observations with the 95% CI excluding 0.5 or AUC > 0.7 indicating a clinically acceptable effect^68^. Similar approaches were described before^69,70^. We also used the AUC approach to test the parameters derived from the 5 s EEG episodes from 600 s before until 600 s after LOvR/LOpR versus the averaged parameter from − 10 to 10 s around LOvR/LOpR, defined as the parameter value at LOvR/LOpR.

## Supplementary information

Supplementary Information.

## Data Availability

The datasets analysed during the current study are available from PS on reasonable request.
