# Supplementary material for: The influence of induction speed on the frontal (processed) EEG
Source: Sci Rep. 2020 Nov 10;10:19444. doi: 10.1038/s41598-020-76323-8 (PMC7655958; doi:10.1038/s41598-020-76323-8)
Supplement: Supplementary file 1 — Supplementary Information. [file 41598_2020_76323_MOESM1_ESM.pdf]

The influence of induction speed on the frontal (processed) EEG

D. P. Obert[1], P. Sepúlveda[2], S. Kratzer[3], G. Schneider[4], M. Kreuzer[5*]

[1] Resident, Technical University Munich, Germany, Klinikum rechts der Isar, Department of Anesthesiology and Intensive Care Medicine; david.obert@tum.de

[2] Associate Professor, Universidad Austral, Chile, Hospital Base San José, Osorno Department of Anesthesiology; pasevou@gmail.com

[3] Attending, Technical University Munich, Germany, Klinikum rechts der Isar, Department of Anesthesiology and Intensive Care Medicine; s.kratzer@tum.de

[4] Chairman, Technical University Munich, Germany, Klinikum rechts der Isar, Department of Anesthesiology and Intensive Care Medicine; gerhard.schneider@tum.de

[5*] Senior Researcher, Technical University Munich, Germany, Klinikum rechts der Isar, Department of Anesthesiology and Intensive Care Medicine; m.kreuzer@tum.de

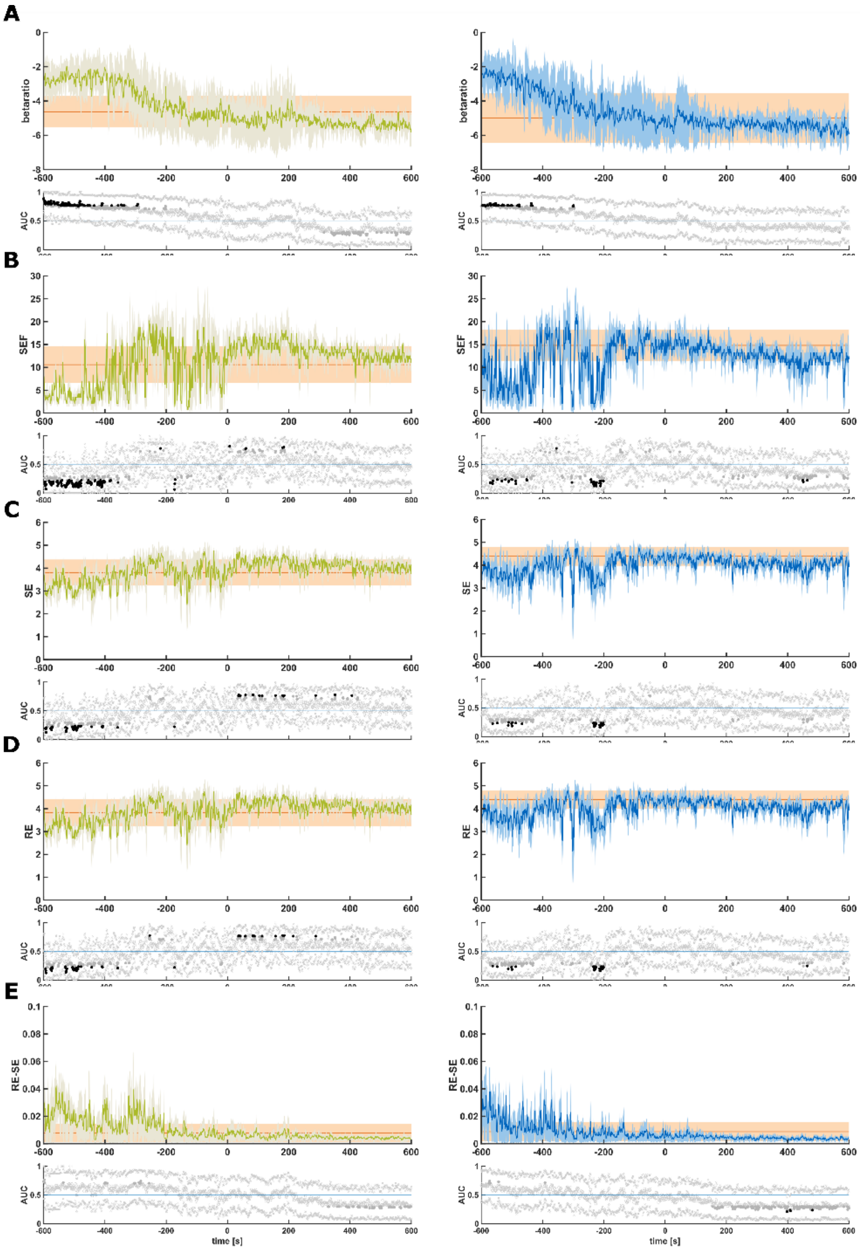

Figure S1: Trend of various EEG parameters of patients with slow induction from 10 min before until 10 min after loss of responsiveness to verbal (LOvR, left) and painful (LOpR) stimulation, compared to the -10 s to 10 s around LOR

A. Beta ratio
B. SEF95
C. SE
D. RE
E. RE-SE

The green (LOvR) or blue (LOpR) line indicates the median parameter of all patients during slow induction. The shaded areas reflect the median absolute deviation. The orange line indicates the median at LOvR or LOpR and the light orange the median absolute deviation.

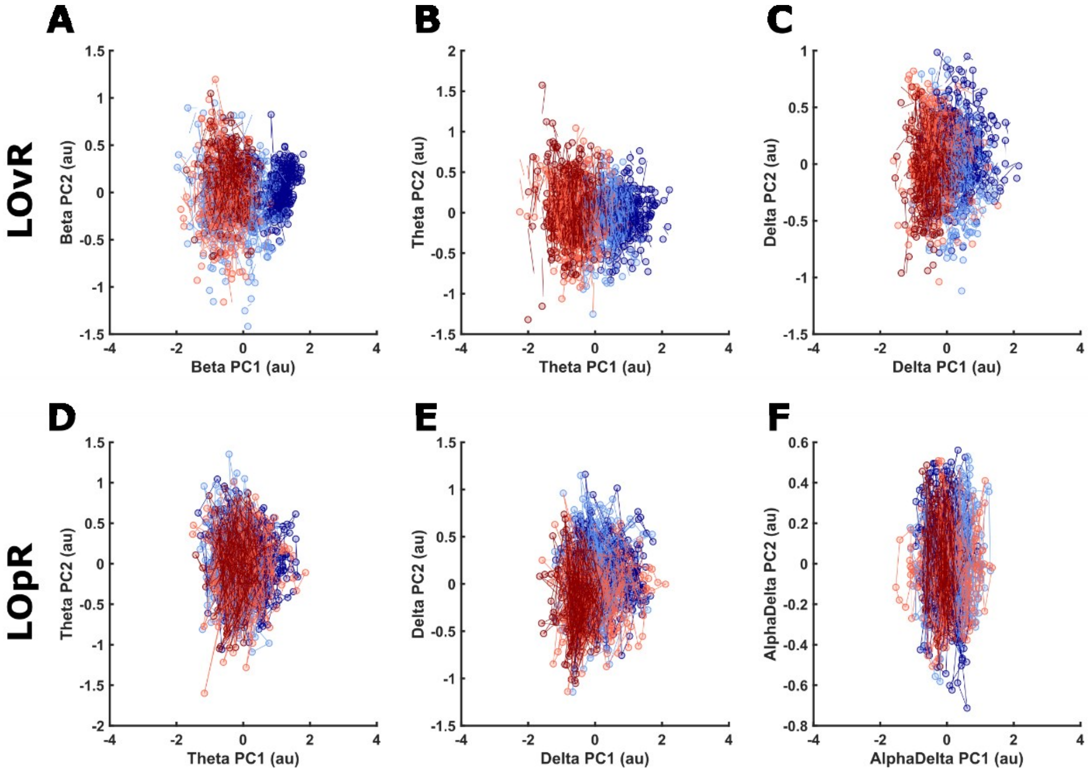

Figure S2: Visualization of the PCA trajectories with no clear separation for loss of responsiveness to verbal (LOvR, left) and painful (LOpR) stimulation:

A. LOvR: beta
B. LOvR: theta
C. LOvR: delta
D. LOpR: theta
E. LOpR: delta
F. LOpR: alpha-delta
